# An ATAC-seq Dataset Uncovers the Regulatory Landscape During Axolotl Limb Regeneration

**DOI:** 10.3389/fcell.2021.651145

**Published:** 2021-03-30

**Authors:** Xiaoyu Wei, Hanbo Li, Yang Guo, Xiaoying Zhao, Yang Liu, Xuanxuan Zou, Li Zhou, Yue Yuan, Yating Qin, Chunyan Mao, Guodong Huang, Yeya Yu, Qiuting Deng, Weimin Feng, Jiangshan Xu, Mingyue Wang, Shanshan Liu, Huanming Yang, Longqi Liu, Chuanyu Liu, Ying Gu

**Affiliations:** ^1^BGI Education Center, University of Chinese Academy of Sciences, Shenzhen, China; ^2^BGI-Shenzhen, Shenzhen, China; ^3^BGI-Qingdao, BGI-Shenzhen, Qingdao, China; ^4^BGI College, Zhengzhou University, Zhengzhou, China; ^5^James D. Watson Institute of Genome Sciences, Hangzhou, China; ^6^Shenzhen Bay Laboratory, Shenzhen, China; ^7^Guangdong Provincial Key Laboratory of Genome Read and Write, BGI-Shenzhen, Shenzhen, China

**Keywords:** limb, regenertion, axolotl, ATAC-seq, regulatory element

## Introduction

Tissue regenerative potential varies significantly across species, tissues, and ages (Yun, 2015; Iismaa et al., 2018). For example, planarian can reconstruct its whole body from small fragments of the original organism (Pellettieri et al., 2010; Zeng et al., 2018); in contrast, many vertebrate organs, such as the heart, can only regenerate primarily through preexisting proliferating cardiomyocytes, like in adult zebrafish and neonatal mice (Vivien et al., 2016). Since Spallanzani first reported the salamander regeneration in 1760s, scientists have been devoted to decipher the codes of such powerful regenerative capability (Dinsmore, 1991). Using different methods to analyze the cellular and molecular phenomena during salamander limb or tail regeneration, researchers revealed complex processes including clotting, immune activation, apoptosis, and reprogramming (Tanaka, 2016). Within such process, a mass of cells called blastema proliferates from the wounded site and fully regenerates the lost body part (McCusker et al., 2015; Haas and Whited, 2017).

Axolotl (*Ambystoma mexicanum*) is a species of salamander, which has been used as the model animal to investigate key biological processes such as embryo development, limb regeneration, and central nervous system regeneration for nearly 150 years (Pietsch, 1961; Schreckenberg and Jacobson, 1975; Seyedhassantehrani et al., 2017). Although several studies have focused on bulk transcriptome studies (Monaghan et al., 2009; Campbell et al., 2011; Knapp et al., 2013; Stewart et al., 2013; Wu et al., 2013; Bryant et al., 2017), the axolotl genome was not assembled until 2018 with features of large sizes (32 Gb) and abundant repetitive sequences (Nowoshilow et al., 2018). Interestingly, in axolotl, intron size expands 13- to 25-fold in non–developmentally related orthologous genes and 6- to 11-fold in developmentally related orthologous genes as compared to human, mouse, and frog, thus indicating that a more complex regulatory network in non-coding regions may play an important role in both development and regeneration (Nowoshilow et al., 2018). Since the first axolotl genome assembly, multiple studies have been carried out to investigate the transcriptomic patterns of axolotl limb regeneration at single-cell resolution (Gerber et al., 2018; Leigh et al., 2018; Qin et al., 2020). These studies used single-cell gene expression variations to reflect dynamic cell population changes and cell fate transitions, as well as unique immune responses during regeneration (Tsai et al., 2019; Li et al., 2020; Rodgers et al., 2020). Standing on the shoulder of these studies and looking forward, analysis of the epigenetic regulations, which are responsible for the dynamic gene expression changes, will help scientists to better understand the underlying mechanisms of the regenerative process.

To identify crucial regulatory elements and transcription factors (TFs) that drive or support the regenerative response, the assay for transposase-accessible chromatin using sequencing (ATAC-seq) has been used to profile the chromatin accessibility dynamics in multiple species (Buenrostro et al., 2015). For instance, a genome-wide scan for TF binding motifs in thousands of chromatin regions revealed by ATAC-seq highlighted the role of EGR (early growth response) as a pioneer factor to directly activate regeneration-related genes in *Hofstenia* (Gehrke et al., 2019). In addition to TFs, enhancers also have great significance in regeneration. The conserved teleost regeneration response enhancers in zebrafish and African killifish (*Nothobranchius furzeri*) were uncovered by histone H3K27ac chromatin immunoprecipitation sequencing (ChIP-seq, a marker for active enhancers), bulk RNA sequencing (RNA-seq), and single-cell RNA sequencing (scRNA-seq) (Wang et al., 2020). These studies suggested that epigenetic regulatory elements play fundamental roles in regeneration. However, how the non-coding axolotl genome responds to wounding to regulate gene expression and consequently drive the process of limb regeneration remains to be elucidated.

**Figure 1:**
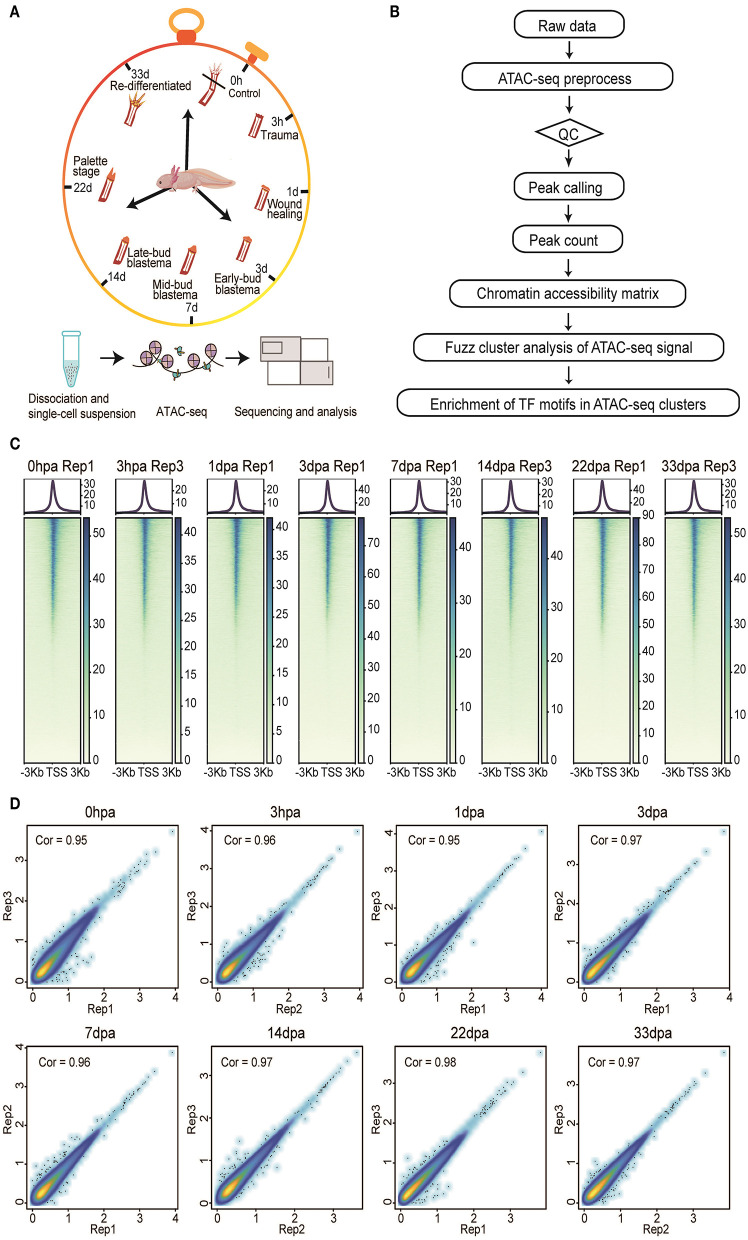
Overview of the experimental, data analysis workflow, and ATAC-seq data quality metrics. **(A)** Three biological replicates (*n* > 3) from eight stages of the axolotl limb regeneration process were collected for ATAC-seq profiling. **(B)** The analysis workflow for ATAC-seq profiles. **(C)** The ATAC-seq signal enrichment around the transcription start sites (TSSs) for eight representative samples. **(D)** Scatter plots showing the Pearson correlations between biological replicates.

Here, we present a comprehensive dataset of chromatin accessibility for eight stages of the axolotl limb regeneration process, including homeostatic [uninjured control, 0 h after amputation (0 hpa)], trauma (3 hpa), wounding healing (1 day after amputation, 1 dpa), early-bud blastema (3 dpa), midbud blastema (7 dpa), late-bud blastema (14 dpa), palette stage (22 dpa), and redifferentiated stages (33 dpa) (Figure 1A). These time points represent the main events during axolotl limb regeneration, making this dataset a valuable platform to understand the complex regulatory network from an overall perspective. We generated 24 samples from the eight stages of limb tissues (three biological replicates per group). Systematic analysis of our dataset identified a total of 342,341 peaks, of which 33,604 showed transient dynamic patterns. We further investigated the occupancy of TFs in clusters with different peaks, which may help to explain the activation and manipulation of these regulatory elements during injury response and regeneration process (Figure 1B).

## Materials and Methods

### Sample Collection

The institutional review board approved all experiments in this study on the ethics committee of BGI (permit BGI-IRB 19059). Axolotl breeding, housing, and tissue isolation were performed as previously described (Li et al., 2020). Briefly, we anesthetized the axolotls with 0.2% Tricaine (ethyl 3-aminobenzoate methane sulfonate) before the amputation surgery. The lower forearm tissues were isolated at eight time points including the homeostatic stage (uninjured control, 0 hpa), trauma (3 hpa), wound healing stage (1 dpa), early-bud blastema (3 dpa), midbud blastema (7 dpa), late-bud blastema (14 dpa), palette (22 dpa), and redifferentiation stage (33 dpa), with three replicates for each stage. These eight time points represent the main phases of axolotl limb regeneration. All tissues were washed with amphibian phosphate-buffered saline for three times before further operation. Tissues were enzymatically digested to cell suspension using 0.2% collagenase type I (BBI, cat. #A004194-0001) and 0.2% collagenase type II (BBI, cat. #A004174-0001) at room temperature for 1 h.

### ATAC-seq Library Preparation and Sequencing

Tissues were transferred to the bottom of the Dounce Homogenizer and dounced within 1 mL 1 × Homogenization Buffer Stable Master Mix until resistance goes away (~30 strokes). The cells were then passed through a 100-μm strainer into a clean Dounce Homogenizer and dounced again for 20 strokes. Nuclei were collected through a 40-μm strainer and counted. Around 50,000 nuclei were transferred into a tube containing 1 mL wash buffer (ATAC-RSB+0.1% Tween-20), and then the samples were centrifuged at 500 rcf at 4°C for 5 min. Transposition reaction was performed as the Omni-ATAC-seq method (Corces et al., 2017). Nuclei were then transferred into 50 μL transposition reaction mixture containing 10 μL of 5 × TAG buffer (BGI, cat. #BGE005B01), 2.5 μL of transposase (100 nM final, BGI, cat. #BGE005), 31.5 μL of PBS, 0.5 μL of 1% digitonin, 0.5 μL of 10% Tween-20, and 5 μL of H_2_O for 30 min at 37°C in a thermomixer by 1,000 rpm.

The transposed DNA was purified with a DNA MinElute kit (Qiagen, Germany) and eluted with 20 μL nuclease-free H_2_O. The purified DNA was amplified for eight cycles using a reaction mixture containing 2.5 μL of Tn5 Ad153 N5 primer (20 μM), 2.5 μL of Tn5 Ad153 N7 primer (20 μM), 25 μL of NEB Next High-Fidelity 2 × polymerase chain reaction (PCR) Master Mix, with a PCR protocol of 72°C for 5 min, 98°C for 30 s, and then eight cycles of 98°C for 10 s, 63°C for 30 s,72°C for 1 min, finally by 72°C for 10 min and hold at 4°C. The 300- to 500-bp size PCR product was selected using AMPure XP beads (Agencourt, cat. #A63882) according to the manual. All libraries were further prepared based on BGISEQ-500 sequencing platform with pair-end 50-bp read length (Huang et al., 2017).

### Preprocessing of the ATAC-seq Datasets

The data of ATAC-seq were trimmed with SOAPnuke (Chen et al., 2018), and reads were aligned to axolotl genome (Nowoshilow et al., 2018) (https://www.axolotl-omics.org/assemblies) by using Sentieon bwa mem (parameter: -K 100,000,000 -M -t 40) (Li, 2013). Subsequently, we filtered out reads with mapping quality of <30. PCR duplicate reads were discarded by applying Picard's MarkDuplicates (http://broadinstitute.github.io/picard/) (Picard Toolkit, 2019). We next performed model-based analysis of ChIP-seq (MACS2) to identify the peak regions with options -B, -q 0.01 –nomodel, -f BAM (Zhang et al., 2008). The irreproducible discovery rate (IDR) method was employed to identify reproducible high-quality peaks between each two biological replicates (Li et al., 2011). Peak signal can be visualized in IGV by the Broad Institute (http://software.broadinstitute.org/software/igv/). A standard peak list was established by merging reproducible peaks of each two replicates for each time point. The standard peak count matrix was calculated using the intersect function of BedTools (Quinlan and Hall, 2010).

### Identification of Dynamic Chromatin Accessible Regions

Reads per million mapped reads (RPM) algorithm was used to normalize the raw count matrix (Wei, 2020). Pearson correlations based on the Log10 RPM matrix were used to calculate the coefficients between different biological replicates across every stage. The RPM matrix for biological replicates was aggregated, and peaks with an average of RPM <1 at all time points were removed. Peaks across time with <50 coefficient of variation were filtered out to form a pseudocluster prior to clustering, which reflects the regions with stable accessibility throughout the regeneration. The remaining peaks were then transformed into normalized data using *Z* score method, followed by performing time course c-means fuzzy clustering with a cluster membership cutoff of 0.8 (Kumar and Futschik, 2007).

Relative genomic region was determined by overlapping each peak with features defined in the custom's annotated genes. The distance to transcription start sites (TSSs) was calculated according to the distance between the peak center and the nearest TSSs using the *distanceToNearest* function in GenomicRanges packages (Lawrence et al., 2013).

Functional enrichment of peaks in each cluster with distance to TSSs <10,000 bp was performed by using the clusterProfiler R package (Yu et al., 2012), with a *q*-value threshold of 0.1 for statistical significance.

The findMotifsGenome.pl script of the HOMER software was employed to perform transcript factor enrichment analysis in regeneration dynamic peaks with default settings (Heinz et al., 2010).

### Pseudobulk RNA-seq Analysis

To investigate the correlation between chromatin dynamics and gene expression changes, we took advantage of single-cell RNA-seq data of these eight stages we published previously and calculated the average expression of each gene to construct a pseudobulk gene expression matrix (Li et al., 2020). Correlation analysis was done between the chromatin accessibility of promoters (TSS ± 2 kb) and closest genes' expression.

## Results

### ATAC-seq Quality Control and Reproducibility of Biological Samples

We inspected our ATAC-seq dataset by regularly used statistics, such as the number of total reads, number of mapped reads, percentage of mapped reads, the number of usable reads, the percentage of final usable reads, and the number of peaks (Supplementary Table 1). We generated more than 1,000 million ATAC-seq reads for each replicate on average. Among these reads, we detected strong enrichment around TSSs (Wei, 2020) (Figure 1C). Moreover, size periodicity of the chromatin accessibility fragments corresponding to integer multiples of nucleosomes (Wei, 2020) demonstrated the reliability of our dataset, this being consistent with previously published ATAC-seq profiles (Buenrostro et al., 2013) (Supplementary Figure 1).

To assess the reproducibility of chromatin accessibility regions between biological replicates, we used the IDR method to filter peaks that overlapped between replicates in each regeneration stage. Pearson correlations based on the Log_10_ RPM matrix were used to calculate the coefficients, showing that a correlation coefficient is higher than 0.9 between each two replicates in each stage, with the exception of replicate 1 from 3 hpa, which was removed for downstream analysis (Figure 1D).

### Temporal Dynamics of Chromatin Accessibility During Regeneration

**Figure 2:**
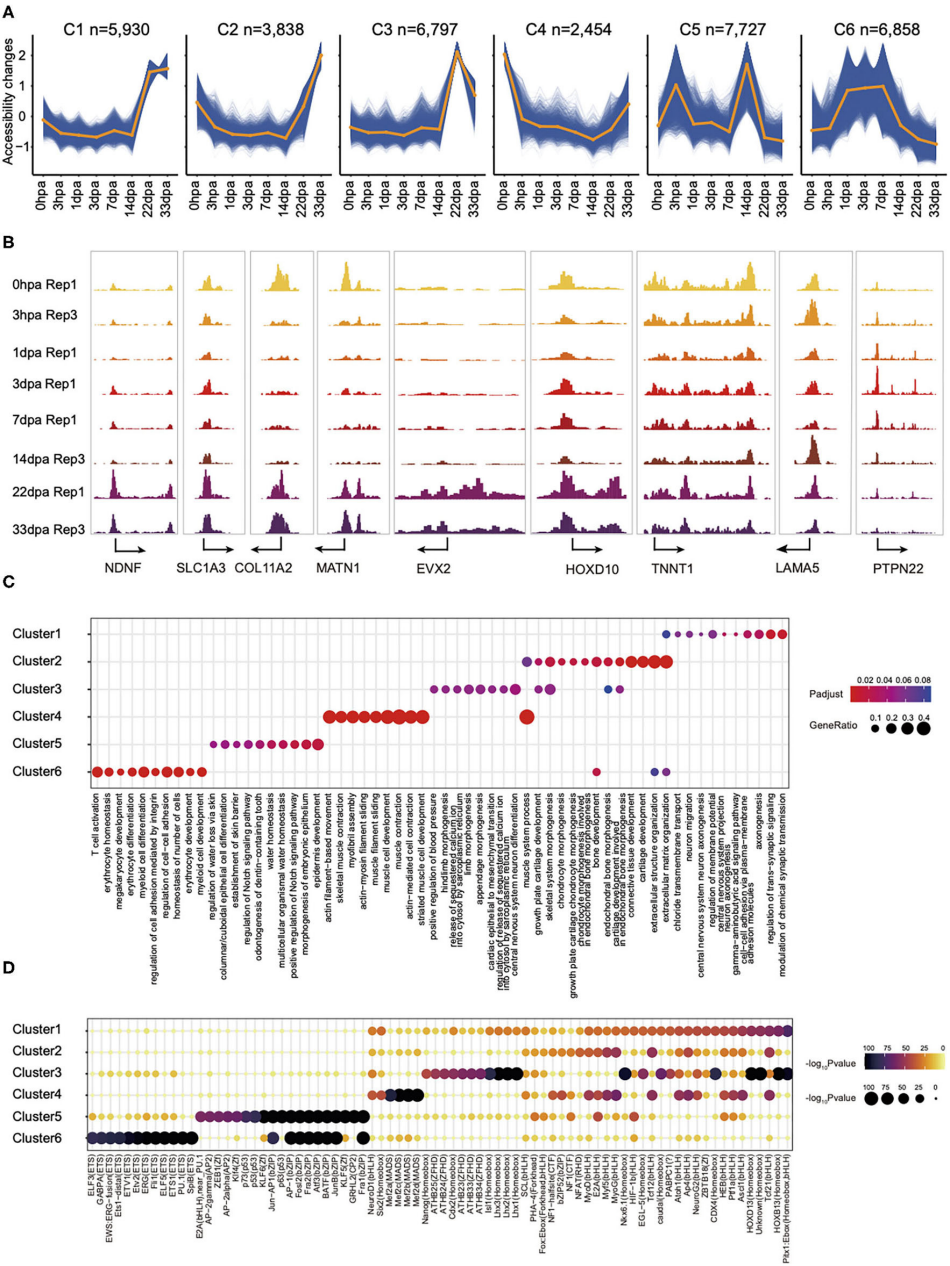
The landscape of chromatin accessibility dynamic changes in axolotl limb regeneration and potential function of dynamic elements identification. **(A)** Fuzzy cluster analysis of ATAC-seq signal. Line plots show standardized ATAC-seq signal, with individual blue lines representing individual loci and the orange line representing the cluster center's values. **(B)** Genome browser views of ATAC-seq signal for the dynamic peaks. **(C)** Enrichment of GO terms in ATAC-seq clusters. Where a point is present, a significant enrichment for the go term of biological process (*x* axis) was found in the ATAC-seq clusters (*y* axis). Point size represents the gene ratio in the go term, and color represents the adjusted *P*-value. **(D)** Enrichment of the indicated TF motifs in each ATAC-seq cluster. The size and color of each point represent the motif enrichment *P*-value (–log10 *P*-value).

To explore the chromatin accessibility with temporal dynamic features during regeneration, we used normalized ATAC-seq read counts in peaks to perform time-course fuzzy clustering. This approach yielded six separated clusters, which indicate six distinct categories defined by regions: (1) those that become accessible transiently at 22- and 33-dpa stages, cluster 1 (C1, *n* = 5,930); (2) regions that are close in the intermediate period of regeneration but accessible after 14 dpa, cluster 2 (C2, *n* = 3,838); (3) regions in which accessibility is established only at 22-dpa stage, cluster 3 (C3, *n* = 6,797); (4) regions accessible in the control but that exhibit loss of accessibility shortly at 3 hpa and later stages, cluster 4 (C4, *n* = 2,454); (5) regions that are accessible in specifically at 3 hpa and 14 dpa, cluster 5 (C5, *n* = 7,727); (6) regions that are stably accessible at the intermediate stages of regeneration, cluster 6 (C6, *n* = 6,858). This clustering highlighted several characteristics of chromatin reconfiguration during regeneration (Figure 2A). These data collectively demonstrated that the chromatin state is remodeling rapidly in the first few hours following amputation, to prepare for the subsequent regeneration process. The dynamics of chromatin accessibility provides a new perspective to understand the cell fate decision in axolotl limb regeneration process.

Peaks in C1 are highly enriched in Gene Ontology (GO) terms related to axonogenesis. Examples of C1 include a promoter in the *Ndnf* locus, which is a novel neurotrophic factor derived from neurons that may be useful in the treatment of neuronal degeneration diseases and nerve injuries (Kuang et al., 2010). C2 consists of elements that are highly accessible in the extracellular matrix organization and connective tissue development. For example, *Col11a2*, a fibril-forming collagen found mainly in the cartilage extracellular matrix, is important for the integrity and development of the skeleton (Lui et al., 1996). We also found some genes associated to limb morphogenesis in C3 such as the Hoxbox gene, *Evx2*, and *Hoxd10* (Herault et al., 1996; Tarchini and Duboule, 2006). GO terms enriched in C4 were related to muscle cell development, whereas those in C5 were related to epidermis development. Interestingly, we observed some immune response–related GO terms in C6, such as T-cell activation and myeloid cell differentiation (Figures 2B,C), which is consistent with the inflammatory process following injury (Supplementary Figure 2).

### TF Enrichment of Dynamic *cis*-Regulatory Elements

Our analysis also indicated that the binding site for TF that bound to C1 is enriched for NeuroG2 (Figure 2D). NeuroG2 is a TF that can specify a neuronal fate and expressed in neural progenitor cells within the developing central and peripheral nervous systems (Dennis et al., 2019). Notably, we also observed major binding events for the pioneer factor PU.1 in C6, this being a master transcriptional regulator in activating many target genes during both myeloid and B-lymphoid development (Turkistany and DeKoter, 2011). In addition, transcript factors from the MyoG, MyoD, and Mef families, which are essential for the development of functional skeletal muscle, were found in C4 (Al-Khalili et al., 2004; Ganassi et al., 2020). Taken together, we provide a high-quality comprehensive dataset to study the regenerative epigenomic dynamics of axolotl limb regeneration.

## Conclusions

To summarize, by applying the state-of-the-art technique ATAC-seq, we provide the first chromatin accessibility landscape in axolotl regenerative limb tissues from the immediate response stage to the complete recovery stage. These data will be of great importance to the studies of various scientific disciplines such as development, cell reprogramming, and mechanisms underlying regeneration. Further analysis of these datasets by focusing on the differentially regulated regions may help deduce key regulatory elements that are critical for regeneration initiation in the axolotl limb in the future.

## Data Availability Statement

All raw data have been submitted to the CNGB (Nucleotide Sequence Archive) (https://db.cngb.org/search/project/CNP0001445/), and also the NCBI (SRA) (https://www.ncbi.nlm.nih.gov/search/all/?term=PRJNA682840).

## Ethics Statement

The animal study was reviewed and approved by the Ethics Committee of BGI.

## Author Contributions

XW, YGu, HL, CL, YGuo, and XZh conceived the idea. YGuo and XZh collected samples. XZh and YGuo generated the data. HL, YYua, YQ, CM, JX, MW, YYu, and QD assisted with the experiments. XW analyzed the data with the assistance from YL, XZo, LZ, GH, and WF. XW wrote the manuscript with the input of HL, YGuo, and XZh. YGu and CL supervised the study and revised the manuscript. LL, HY, and SL provided helpful comments on this study. All authors reviewed and approved the final manuscript.

## Conflict of Interest

The authors declare that the research was conducted in the absence of any commercial or financial relationships that could be construed as a potential conflict of interest.

## Supplementary Material

Supplementary Table 1 ATAC-seq metadata and mapping statistics. ^*^Mapped reads: total number of reads minus number of unaligned reads. ^*^Usable reads: number of mapped reads minus number of low mapping quality and duplicate reads.

Supplementary Figure 1 The insert size distribution of ATAC-seq profiles for the same samples shown in Figure 2A.

Supplementary Figure 2 Decomposition of chromatin accessibility and gene expression dynamics during axolotl limb regeneration. **(A)** Chromatin accessibility dynamics of promoters (TSS ± 2 kb) in six clusters. **(B)** Pseudobulk gene expression in **(A)**. **(C)** Heatmap shows the correlation between bulk ATAC-seq and pseudobulk RNA for each gene in C6. **(D)** Chromatin accessibility and gene expression for genes in C6.

## References

[B1] Al-Khalili L. Chibalin A. V. Yu M. Sjodin B. Nylén C. Zierath J. R.. (2004). MEF2 activation in differentiated primary human skeletal muscle cultures requires coordinated involvement of parallel pathways. Am. J. Physiol. Cell Physiol. 286, C1410–C1416. 10.1152/ajpcell.00444.2003 14960415

[B2] Bryant D. M. Johnson K. DiTommaso T. Tickle T. Couger M. B. Payzin-Dogru D.. (2017). A tissue-mapped axolotl de novo transcriptome enables identification of limb regeneration factors. Cell Rep. 18, 762–776. 10.1016/j.celrep.2016.12.063 28099853 PMC5419050

[B3] Buenrostro J. D. Giresi P. G. Zaba L. C. Chang H. Y. Greenleaf W. J. (2013). Transposition of native chromatin for fast and sensitive epigenomic profiling of open chromatin, DNA-binding proteins and nucleosome position. Nat. Methods 10:1213. 10.1038/nmeth.2688 24097267 PMC3959825

[B4] Buenrostro J. D. Wu B. Chang H. Y. Greenleaf W. J. (2015). ATAC-seq: a method for assaying chromatin accessibility genome-wide. Curr. Protoc. Mol. Biol. 109, 21–29. 10.1002/0471142727.mb2129s109 25559105 PMC4374986

[B5] Campbell L. J. Suárez-Castillo E. C. Ortiz-Zuazaga H. Knapp D. Tanaka E. M. Crews C. M. J. D.D. (2011). Gene expression profile of the regeneration epithelium during axolotl limb regeneration. Dev. Dyn. 240, 1826–1840. 10.1002/dvdy.22669 21648017 PMC3297817

[B6] Chen Y. Chen Y. Shi C. Huang Z. Zhang Y. Li S.. (2018). SOAPnuke: a MapReduce acceleration-supported software for integrated quality control and preprocessing of high-throughput sequencing data. Gigascience 7:gix120. 10.1093/gigascience/gix120 29220494 PMC5788068

[B7] Corces M. R. Trevino A. E. Hamilton E. G. Greenside P. G. Sinnott-Armstrong N. A. Vesuna S.. (2017). An improved ATAC-seq protocol reduces background and enables interrogation of frozen tissues. Nat. Methods 14, 959–962. 10.1038/nmeth.4396 28846090 PMC5623106

[B8] Dennis D. J. Han S. Schuurmans C. (2019). bHLH transcription factors in neural development, disease, and reprogramming. Brain Res. 1705, 48–65. 10.1016/j.brainres.2018.03.013 29544733

[B9] Dinsmore C. E. (1991). A *H*istory of *R*egeneration *Research: Milestones in the Evolution of a Science*. Cambridge: Cambridge University Press.

[B10] Ganassi M. Badodi S. Wanders K. Zammit P. S. Hughes S. M. (2020). Myogenin is an essential regulator of adult myofibre growth and muscle stem cell homeostasis. Elife 9:e60445. 10.7554/eLife.60445.sa2 33001028 PMC7599067

[B11] Gehrke A. R. Neverett E. Luo Y.-J. Brandt A. Ricci L. Hulett R. E.. (2019). Acoel genome reveals the regulatory landscape of whole-body regeneration. Science 363:6173. 10.1126/science.aau6173 30872491

[B12] Gerber T. Murawala P. Knapp D. Masselink W. Schuez M. Hermann S.. (2018). Single-cell analysis uncovers convergence of cell identities during axolotl limb regeneration. Science 362:681. 10.1126/science.aaq0681 30262634 PMC6669047

[B13] Haas B. J. Whited J. L. (2017). Advances in decoding axolotl limb regeneration. Trends Genet. 33, 553–565. 10.1016/j.tig.2017.05.006 28648452 PMC5534018

[B14] Heinz S. Benner C. Spann N. Bertolino E. Lin Y. C. Laslo P.. (2010). Simple combinations of lineage-determining transcription factors prime cis-regulatory elements required for macrophage and B cell identities. Mol. Cell 38, 576–589. 10.1016/j.molcel.2010.05.004 20513432 PMC2898526

[B15] Herault Y. Hraba-Renevey S. Van der Hoeven F. Duboule D. (1996). Function of the Evx-2 gene in the morphogenesis of vertebrate limbs. EMBO J. 15, 6727–6738. 10.1002/j.1460-2075.1996.tb01062.x 8978698 PMC452496

[B16] Huang J. Liang X. Xuan Y. Geng C. Li Y. Lu H.. (2017). A reference human genome dataset of the BGISEQ-500 sequencer. Gigascience 6:gix024. 10.1093/gigascience/gix024 28379488 PMC5467036

[B17] Iismaa S. E. Kaidonis X. Nicks A. M. Bogush N. Kikuchi K. Naqvi N.. (2018). Comparative regenerative mechanisms across different mammalian tissues. NPJ Regen. Med. 3, 1–20. 10.1038/s41536-018-0044-5 29507774 PMC5824955

[B18] Knapp D. Schulz H. Rascon C. A. Volkmer M. Scholz J. Nacu E.. (2013). Comparative transcriptional profiling of the axolotl limb identifies a tripartite regeneration-specific gene program. PLoS ONE 8:e61352. 10.1371/journal.pone.0061352 23658691 PMC3641036

[B19] Kuang X.-L. Zhao X.-M. Xu H.-F. Shi Y.-Y. Deng J.-B. Sun G.-T. (2010). Spatio-temporal expression of a novel neuron-derived neurotrophic factor (NDNF) in mouse brains during development. BMC Neurosci. 11, 1–11. 10.1186/1471-2202-11-137 20969804 PMC2984559

[B20] Kumar L. Futschik M. E. (2007). Mfuzz: a software package for soft clustering of microarray data. Bioinformation 2:5. 10.6026/97320630002005 18084642 PMC2139991

[B21] Lawrence M. Huber W. Pages H. Aboyoun P. Carlson M. Gentleman R.. (2013). Software for computing and annotating genomic ranges. PLoS Comput. Biol. 9:e1003118. 10.1371/journal.pcbi.1003118 23950696 PMC3738458

[B22] Leigh N. D. Dunlap G. S. Johnson K. Mariano R. Oshiro R. Wong A. Y.. (2018). Transcriptomic landscape of the blastema niche in regenerating adult axolotl limbs at single-cell resolution. Nat. Commun. 9, 1–14. 10.1038/s41467-018-07604-0 30514844 PMC6279788

[B23] Li H. (2013). Aligning sequence reads, clone sequences and assembly contigs with BWA-MEM. arXiv [Preprint]. arXiv:1303.3997. 10.6084/M9.FIGSHARE.963153.V1

[B24] Li H. Wei X. Zhou L. Zhang W. Wang C. Guo Y.. (2020). Dynamic cell transition and immune response landscapes of axolotl limb regeneration revealed by single-cell analysis. Protein Cell 12, 57–66. 10.1007/s13238-020-00763-1 32748350 PMC7815851

[B25] Li Q. Brown J. B. Huang H. Bickel P. J. (2011). Measuring reproducibility of high-throughput experiments. Ann. Appl. Stat. 5, 1752–1779. 10.1214/11-AOAS466

[B26] Lui V. C. Ng L. J. Sat E. W. Cheah K. S. (1996). The human α2 (XI) collagen gene (COL11A2): completion of coding information, identification of the promoter sequence, and precise localization within the major histocompatibility complex reveal overlap with the KE5 gene. Genomics 32, 401–412. 10.1006/geno.1996.0135 8838804

[B27] McCusker C. Bryant S. V. Gardiner D. M. (2015). The axolotl limb blastema: cellular and molecular mechanisms driving blastema formation and limb regeneration in tetrapods. Regeneration 2, 54–71. 10.1002/reg2.32 27499868 PMC4895312

[B28] Monaghan J. R. Epp L. G. Putta S. Page R. B. Walker J. A. Beachy C. K.. (2009). Microarray and cDNA sequence analysis of transcription during nerve-dependent limb regeneration. BMC Biol. 7:1. 10.1186/1741-7007-7-1 19144100 PMC2630914

[B29] Nowoshilow S. Schloissnig S. Fei J.-F. Dahl A. Pang A. W. Pippel M.. (2018). The axolotl genome and the evolution of key tissue formation regulators. Nature 554, 50–55. 10.1038/nature25458 29364872

[B30] Pellettieri J. Fitzgerald P. Watanabe S. Mancuso J. Green D. R. Alvarado A. S. (2010). Cell death and tissue remodeling in planarian regeneration. Dev. Biol. 338, 76–85. 10.1016/j.ydbio.2009.09.015 19766622 PMC2835816

[B31] Picard Toolkit (2019). Broad Institute, GitHub Repository. Available online at: http://broadinstitute.github.io/picard/

[B32] Pietsch P. (1961). Differentiation in regeneration I. The development of muscle and cartilage following deplantation of regenerating limb blastemata of Amblystoma larvae. Dev. Biol. 3, 255–264. 10.1016/0012-1606(61)90046-X 13735628

[B33] Qin T. Fan C.-m. Wang T.-z. Sun H. Zhao Y.-y. Yan R.-j.. (2020). Single-cell RNA-seq reveals novel mitochondria-related musculoskeletal cell populations during adult axolotl limb regeneration process. Cell Death Diff. 28, 1110–1125. 10.1038/s41418-020-00640-8 33116295 PMC7937690

[B34] Quinlan A. R. Hall I. M. (2010). BEDTools: a flexible suite of utilities for comparing genomic features. Bioinformatics 26, 841–842. 10.1093/bioinformatics/btq033 20110278 PMC2832824

[B35] Rodgers A. K. Smith J. J. Voss S. R. (2020). Identification of immune and non-immune cells in regenerating axolotl limbs by single-cell sequencing. Exp. Cell Res. 394:112149. 10.1016/j.yexcr.2020.112149 32562784 PMC7483677

[B36] Schreckenberg G. Jacobson A. (1975). Normal stages of development of the axolotl, Ambystoma mexicanum. Dev. Biol. 42, 391–399. 10.1016/0012-1606(75)90343-7 1167837

[B37] Seyedhassantehrani N. Otsuka T. Singh S. Gardiner D. M. (2017). The axolotl limb regeneration model as a discovery tool for engineering the stem cell niche. Curr. Stem Cell Rep. 3, 156–163. 10.1007/s40778-017-0085-5 29230380 PMC5722022

[B38] Stewart R. Rascón C. A. Tian S. Nie J. Barry C. Chu L. F.. (2013). Comparative RNA-seq analysis in the unsequenced axolotl: the oncogene burst highlights early gene expression in the blastema PLoS Comput. Biol. 9:e1002936. 10.1371/journal.pcbi.1002936 23505351 PMC3591270

[B39] Tanaka E. M. (2016). The molecular and cellular choreography of appendage regeneration. Cell 165, 1598–1608. 10.1016/j.cell.2016.05.038 27315477

[B40] Tarchini B. Duboule D. (2006). Control of Hoxd genes' collinearity during early limb development. Dev. Cell 10, 93–103. 10.1016/j.devcel.2005.11.014 16399081

[B41] Tsai S. L. Baselga-Garriga C. Melton D. A. (2019). Blastemal progenitors modulate immune signaling during early limb regeneration. Development 146:e169128. 10.1242/dev.169128 30602532

[B42] Turkistany S. A. DeKoter R. P. (2011). The transcription factor PU. 1 is a critical regulator of cellular communication in the immune system. Arch. Immunol. Ther. Exp. 59, 431–440. 10.1007/s00005-011-0147-9 21972017

[B43] Vivien C. J. Hudson J. E. Porrello E. R. (2016). Evolution, comparative biology and ontogeny of vertebrate heart regeneration. NPJ Regen. Med. 1, 1–14. 10.1038/npjregenmed.2016.12 29302337 PMC5744704

[B44] Wang W. Hu C.-K. Zeng A. Alegre D. Hu D. Gotting K.. (2020). Changes in regeneration-responsive enhancers shape regenerative capacities in vertebrates. Science 369:3090. 10.1126/science.aaz3090 32883834 PMC9479427

[B45] Wei X. (2020). An ATAC-seq Dataset Uncovers the Regulatory Landscape Axolotl Limb Regeneration. Available online at: 10.6084/m9.figshare.13370468.v1 PMC8044901 33869207

[B46] Wu C. H. Tsai M. H. Ho C. C. Chen C. Y. Lee H. S. J. B.G. (2013). De novo transcriptome sequencing of axolotl blastema for identification of differentially expressed genes during limb regeneration. BMC Genomics 14:434. 10.1186/1471-2164-14-434 23815514 PMC3702472

[B47] Yu G. Wang L.-G. Han Y. He Q.-Y. (2012). clusterProfiler: an R package for comparing biological themes among gene clusters. Omics 16, 284–287. 10.1089/omi.2011.0118 22455463 PMC3339379

[B48] Yun M. H. (2015). Changes in regenerative capacity through lifespan. Int. J. Mol. Sci. 16, 25392–25432. 10.3390/ijms161025392 26512653 PMC4632807

[B49] Zeng A. Li H. Guo L. Gao X. McKinney S. Wang Y.. (2018). Prospectively isolated tetraspanin(+) neoblasts are adult pluripotent stem cells underlying planaria regeneration. Cell 173, 1593–1608. 10.1016/j.cell.2018.05.006 29906446 PMC9359418

[B50] Zhang Y. Liu T. Meyer C. A. Eeckhoute J. Johnson D. S. Bernstein B. E.. (2008). Model-based analysis of ChIP-Seq (MACS). Genome Biol. 9, 1–9. 10.1186/gb-2008-9-9-r137 PMC2592715 18798982

