# Supplementary material for: An ATAC-seq Dataset Uncovers the Regulatory Landscape During Axolotl Limb Regeneration
Source: Front Cell Dev Biol. 2021 Mar 30;9:651145. doi: 10.3389/fcell.2021.651145 (PMC8044901; doi:10.3389/fcell.2021.651145)
Supplement: Supplementary Table 1 — ATAC-seq metadata and mapping statistics. *Mapped reads: total number of reads minus number of unaligned reads. *Usable reads: number of mapped reads minus number of low mapping quality and duplicate reads. [file Data_Sheet_1.ZIP › Additional file/Supplementary Table 1.pdf]

Sample ID	Total Reads	Mapped Reads	Mapping Ratio	Usable Reads	Percentage of Usable Reads	Number of Peaks
0hpa_rep1	1,330,625,204	1,267,152,383	0.95	708,227,174	0.56	181,387
0hpa_rep2	1,268,782,886	1,226,528,554	0.97	716,782,157	0.58	187,077
0hpa_rep3	1,204,478,674	1,163,692,721	0.97	677,985,596	0.58	191,640
3hpa_rep1	1,343,183,186	1,178,162,441	0.88	640,058,889	0.54	122,083
3hpa_rep2	1,481,928,278	1,402,775,606	0.95	772,087,751	0.55	156,383
3hpa_rep3	1,291,170,100	1,228,250,948	0.95	667,930,219	0.54	125,741
1dpa_rep1	1,503,287,972	1,389,128,578	0.92	681,687,741	0.49	112,129
1dpa_rep2	1,220,628,276	1,156,663,775	0.95	595,249,723	0.51	72,145
1dpa_rep3	1,197,076,154	1,128,694,781	0.94	572,845,086	0.51	86,152
3dpa_rep1	1,528,378,436	1,415,711,343	0.93	759,307,888	0.54	198,035
3dpa_rep2	1,515,122,874	1,433,525,556	0.95	764,990,007	0.53	210,508
3dpa_rep3	1,490,849,480	1,402,822,875	0.94	764,215,041	0.54	214,135
7dpa_rep1	1,269,838,340	1,192,376,009	0.94	594,919,142	0.50	134,003
7dpa_rep2	1,120,775,202	1,061,904,454	0.95	520,705,835	0.49	119,253
7dpa_rep3	1,316,251,554	1,248,622,111	0.95	668,990,976	0.54	129,455
14dpa_rep1	1,491,704,698	1,433,712,190	0.96	856,513,753	0.60	228,328
14dpa_rep2	1,373,505,032	1,315,392,781	0.96	783,596,088	0.60	191,726
14dpa_rep3	1,519,886,954	1,461,196,642	0.96	870,803,779	0.60	237,613
22dpa_rep1	1,538,676,850	1,415,282,683	0.92	778,929,692	0.55	300,151
22dpa_rep2	1,235,113,852	1,177,767,758	0.95	678,018,606	0.58	179,514
22dpa_rep3	1,279,892,132	1,228,506,596	0.96	733,296,331	0.57	245,580
33dpa_rep1	1,336,855,156	1,255,088,901	0.94	659,769,690	0.53	227,484
33dpa_rep2	1,291,796,200	1,228,062,468	0.95	678,434,846	0.55	209,584
33dpa_rep3	1,322,151,260	1,275,453,358	0.96	657,630,156	0.52	188,449
