# Supplementary figures and images for: An ATAC-seq Dataset Uncovers the Regulatory Landscape During Axolotl Limb Regeneration
Source: Front Cell Dev Biol. 2021 Mar 30;9:651145. doi: 10.3389/fcell.2021.651145 (PMC8044901; doi:10.3389/fcell.2021.651145)

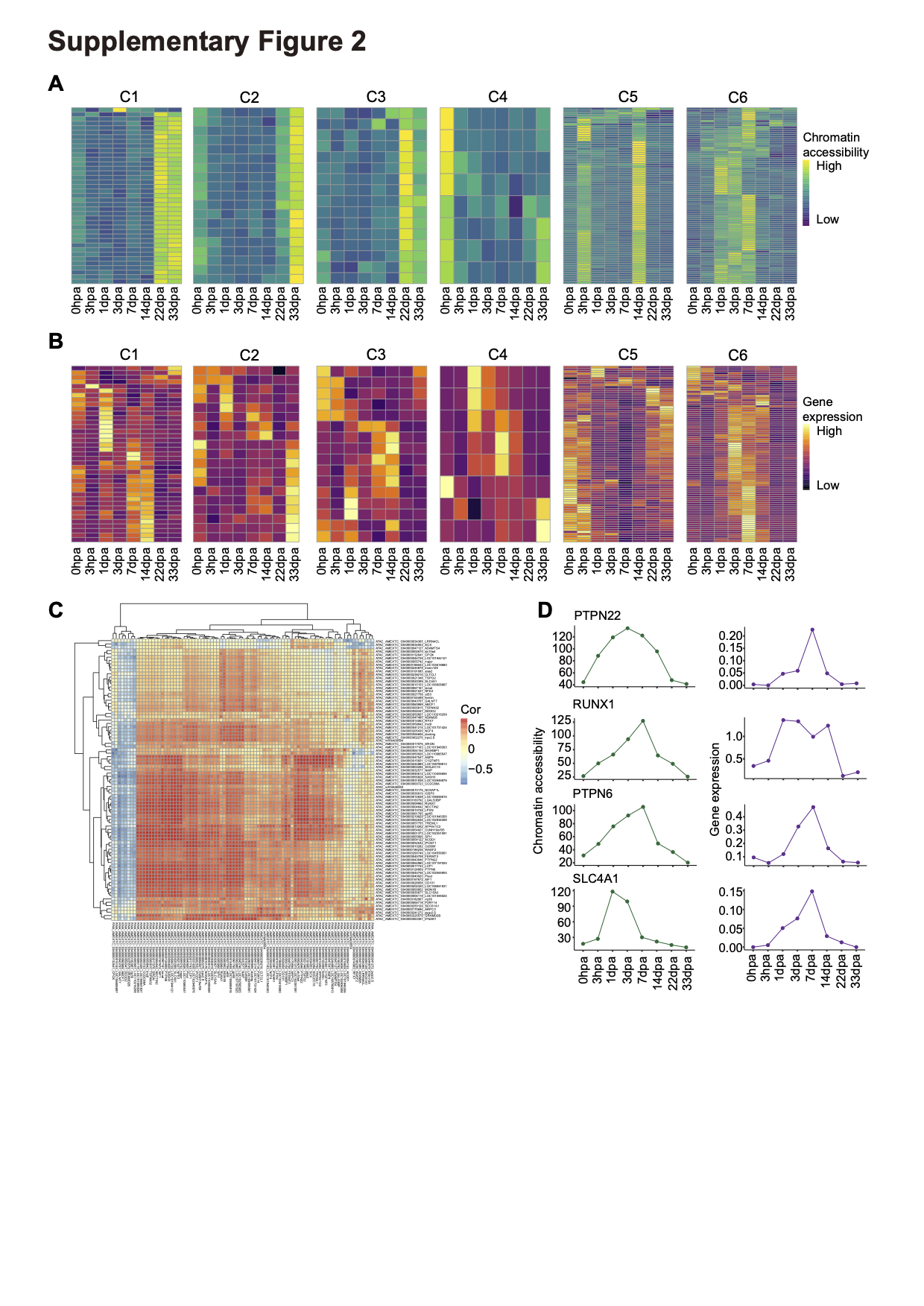

Supplement: Supplementary Table 1 — ATAC-seq metadata and mapping statistics. *Mapped reads: total number of reads minus number of unaligned reads. *Usable reads: number of mapped reads minus number of low mapping quality and duplicate reads. [file Data_Sheet_1.ZIP › Additional file/Supplementary figure 2.tiff]

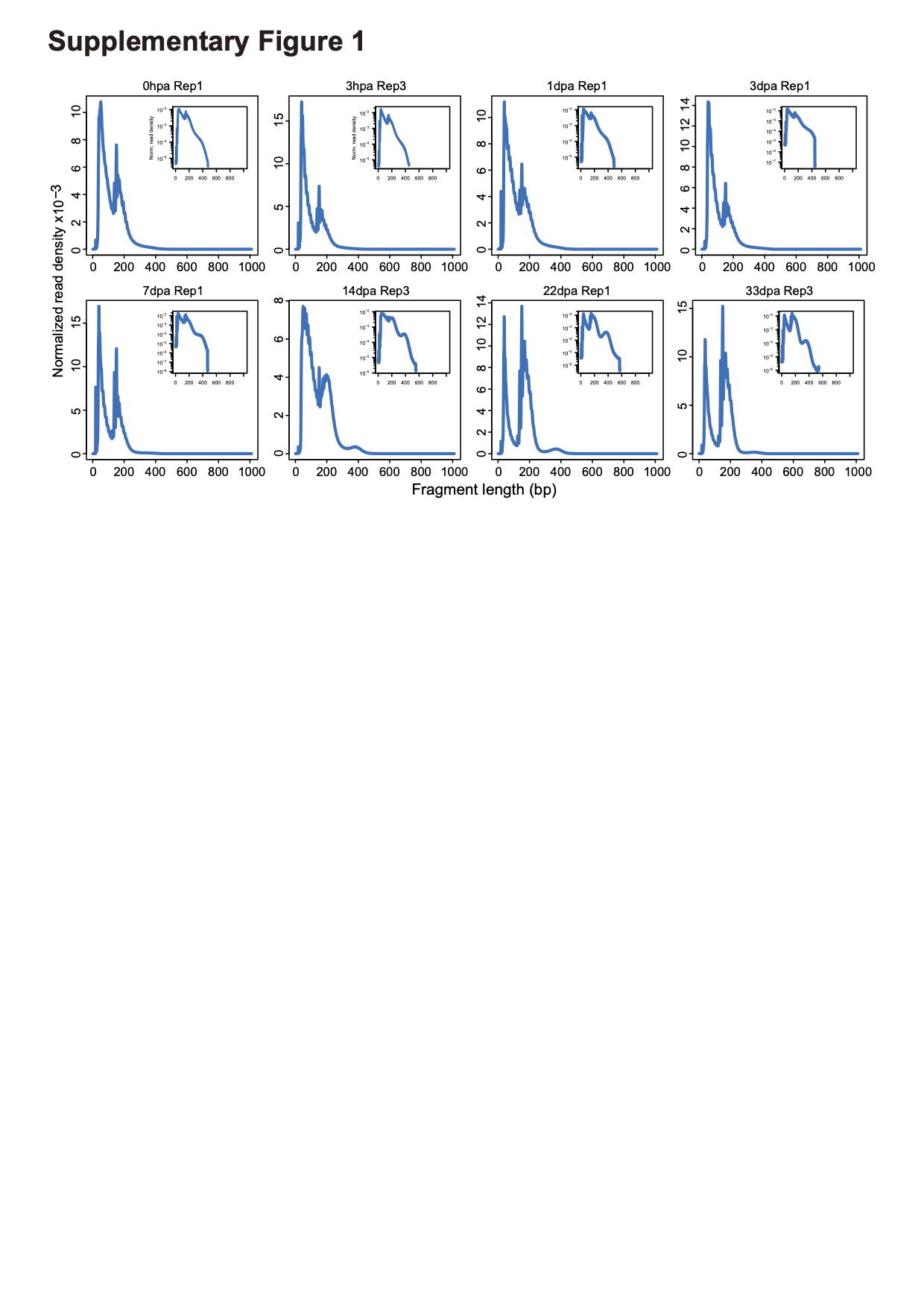

Supplement: Supplementary Table 1 — ATAC-seq metadata and mapping statistics. *Mapped reads: total number of reads minus number of unaligned reads. *Usable reads: number of mapped reads minus number of low mapping quality and duplicate reads. [file Data_Sheet_1.ZIP › Additional file/Supplementary figure 1.tiff]
